# A decade of triage with the Manchester Triage System-The MTS big data study

**DOI:** 10.1371/journal.pone.0344598

**Published:** 2026-04-08

**Authors:** Ingo Gräff, Moritz Berger, Matthias Schmid, Monika Kogej

**Affiliations:** 1 Department of Clinical Acute and Emergency Medicine, University Hospital Bonn, Bonn, Germany; 2 Institute for Medical Biometry, Informatics and Epidemiology, University Hospital Bonn, Bonn, Germany; 3 Core Facility Biostatistics, Central Institute of Mental Health, Medical Faculty Mannheim, Heidelberg University, Mannheim, Germany; Danube Private University, AUSTRIA

## Abstract

**Background:**

Over the past 25 years, numerous studies have evaluated triage system performance, but longitudinal data are limited. This study is the first to assess the Manchester Triage System (MTS) over a decade to determine the accuracy of initial patient assessments and system stability under external influences such as staff turnover, health policy changes, demographic shifts, and the COVID-19 pandemic.

**Methods:**

We conducted a large-scale, monocentric, retrospective observational study at a tertiary care hospital.

**Results:**

Between 2014 and 2023 a total of 355,135 emergency patients were included. Average age increased from 48.1 to 52.1 years. The distribution of the five MTS urgency levels remained stable. Prediction of hospital admission (AUC 0.719–0.756) and ICU admission (AUC 0.831–0.862) was consistent throughout the period. Twelve-hour survival probabilities were above 0.99 for “blue” to “orange” patients and above 0.89 for “red” patients. Short-term mortality prediction (AUC) ranged from 0.845 to 0.894. Thirty-day survival remained >0.8 for “blue” to “orange” stages, while the “red” stage declined from 0.63 (2014) to 0.44 (2023). The AUC for 30-day mortality increased from 0.672 to 0.750 over the decade.

**Conclusion:**

Construct validity assessment demonstrates that the MTS reliably assigns appropriate urgency levels to incoming emergency patients. Longitudinal evaluation shows that its accuracy remained stable over ten years despite high staff turnover, health policy and demographic changes, and the COVID-19 pandemic. These findings confirm the robustness and reliability of the MTS in a high-volume tertiary care setting and support its continued use for emergency patient prioritization.

## Background

Mistriage has consequences both for the individual patient and the performance of the emergency department (ED) as a whole [1]. Therefore, accurate initial triage is a crucial step at the beginning of the ED care pathway. The Manchester Triage System (MTS) is the most frequently used triage system in emergency departments in Germany and large parts of Europe [2,3]. Three years after its introduction in Manchester in 1996, the first report on the initial assessment quality of the MTS was published titled “Does the Manchester Triage System detect the critically ill?” [4]. The colleagues from United Kingdom used admission to the intensive care unit as an evaluation criterion for the MTS and were able to demonstrate that 67% of ICU patients were assigned the urgency level “red” or “orange”. Their conclusion was that the MTS is a sensitive tool for identifying patients who are critically ill upon arrival at the emergency department. They saw potential for improvement in the performance of the MTS primarily through continuous staff training, rather than making changes to the system itself.

In 2014, a German working group evaluated the quality of the German version of the Manchester Triage System for the first time, using suitable criteria on around 45,000 emergency patients over a two-year period. By determining the area under the curve (AUC) of the receiver operating characteristic (ROC) curves of surrogate parameters (inpatient admission/mortality), the authors demonstrated that the accuracy of the MTS was high. This 2014 study showed that the German MTS works well as a triage priority assessment tool for a “first assessment” of emergency patients in the emergency department [5].

Beyond its primary function of rapidly and reliably identifying critically ill patients among newly arriving emergencies, triage serves several additional purposes in the emergency department. It helps to structure and standardize clinical workflows, supports resource planning (e.g., staffing and diagnostics), and contributes to quality assurance and process control. Given the increasing challenges faced by emergency departments—such as overcrowding, shortages of skilled staff, reduced inpatient capacity, and growing health policy requirements—ongoing validation of triage systems, including the MTS, remains essential. The criteria for validity, reliability (examiner or interrater) and accuracy must be differentiated. Accuracy determines whether the triage system measures accurately, specifically how high the rate of over-, under- or incorrect triage is [6]. The accuracy of the MTS is expressed in scientific studies using criteria including sensitivity, specificity, likelihood ratio, negative predictive value (NPV), diagnostic odds ratio and the area under the receiver operator characteristic curve (ROC).

Over the last 25 years, a large number of studies have been published determining the quality of triage systems. A working group consisting of triage specialists conducted a systematic review and meta-analysis to determine the performance of triage systems for identification of high and low urgency patients in the emergency department. Their analysis shows that many individual studies have been performed with different methodologies and that even in studies evaluating the same triage system, there were large differences in performance. Overall, while the quality of the individual triage systems for identifying patients with high and low urgency was moderate to good, the performance comparison of the studies within a triage system shows great variability [3]. It appears that scientific studies conducted on a large group over a longer period of time contribute further essential insights into triage.

The current study examines the German version of the Manchester Triage System (MTS) over a ten-year period using a large-scale dataset. It allows an evaluation of MTS-based triage in a tertiary care emergency department with a broad spectrum of presenting complaints and varying severity of illness and injury. The current study focuses on two main aspects. First, it assesses the accuracy of the MTS as a triage prioritization tool in more than 350,000 emergency patients. Second, it provides a longitudinal evaluation of MTS performance over a decade, taking into account potential exogenous influences such as staff turnover, healthcare policy changes, and demographic developments. In addition, a global pandemic (Covid-19 pandemic) occurred within this 10-year survey period, providing an opportunity to examine the performance of the MTS under the influence of pandemic factors. The current work is therefore intended to make an important scientific contribution to a crucial process in EDs, ultimately aiming to ensure and improve the quality of initial assessment and treatment in the medium to long term.

## Materials and methods

### Setting and study design

This was a single-center retrospective observational study performed at the emergency department of the University Hospital Bonn (UKB), Germany. The UKB is a tertiary hospital with over 38 departments, 31 institutes and 1300 beds, treating approximately 45,000 emergency patients per year. Gynecologic, obstetric, and pediatric emergencies up to age 16 (except for trauma cases in children and children with ear, nose, and throat (ENT) problems) are managed in other nearby departments. Triage in the ED at UKB, performed by the triage nurse, is a standardized process defined according to quality guidelines. Each patient presented as an emergency case is first examined by a specially trained nurse and triaged according to the MTS (triage protocol) [7]. Since the MTS was introduced in the emergency department in 2009, triage has been a daily routine for specialists and the organization since 2014, with the workflow remaining essentially unchanged since then, and therefore did not require an adjustment phase during the study period.

### Manchester Triage System

The MTS uses 53 presentational flow charts for urgency classification (latest physician contact time approved), which are based on the complaints of the emergency patient. Each of these 53 symptom-oriented presentational flow charts is based on an algorithm, which assigns the emergency patient to one of five triage levels according to specific criteria [7]. During the observation period, the quality of triage was regularly evaluated two to three times a year via audit. In addition, between 2014 and 2018, MTS audits were conducted by a physician who was a member of the MTS reference group or by a senior consultant. From 2019 onwards, MTS audits have been performed by a certified MTS trainer. The audit process itself is standardized and defined by the MTS reference group. A random sample of 2% of initial triage assessments is selected and audited with regard to the choice of the presentational flowcharts and the selected discriminator questions. Subsequently, the results are discussed on an individual basis. This process is complemented by specific training measures for the entire team. All nursing staff underwent standardized training in the Manchester Triage System (MTS) as part of the onboarding process, including formal certification by MTS Germany. To maintain consistency and competency in triage application, biannual in-house refresher training sessions were conducted by a locally certified MTS trainer.

The MTS algorithm is applied using a digital protocol that is integrated in the hospital information system (ED Cockpit; Dedalus Health Care Systems Group Bonn, Germany) since 2009.

### Outcome parameters

Since there is no gold standard for assessing the accuracy of triage instruments, against which the assigned triage level of the patient can be compared, it is common practice to work with ‘constructed validity’. This involves using surrogate parameters that allow a statement to be made as to whether the triage instrument reliably assigns a triage level. The current study uses the prediction, which is determined by area under the curve (AUC) of the receiver operating characteristic (ROC) curves and inpatient admission (normal ward/ICU). Since sensitivity is evaluated in relation to specificity, it can be simplified to say that a good prediction can be expected if, for example, only patients classified as more urgent are admitted to the ICU, or patients classified as less urgent are discharged as outpatients. The AUC can take values between 0.5 (50% chance) and 1, with a higher value indicating better quality.

In the current study, 30-day mortality was used as an established outcome parameter in clinical and health services research. It is commonly applied to assess patient course following a defined index event, such as presentation to the emergency department [8,9]. In addition, short-term mortality (death < 12 hours) was examined.

### Data collection

The data considered here were collected over a period of ten years from 2014 to 2023. The data were accessed on May 31, 2024. Each patient is administered before triage and is therefore assigned a unique case number in the hospital information system (HIS). All data directly related to emergency treatment in the ED were taken from the triage protocol such as sex, age, allocation, urgency level, presentational flow charts and discriminators. The triage protocol is an integral part of the patient’s electronic health record. Data resulting from hospital admission, treatment level and outcome (mortality) were extracted from the patient’s electronic health record.

### Inclusion and exclusion criteria

**Fig 1:**
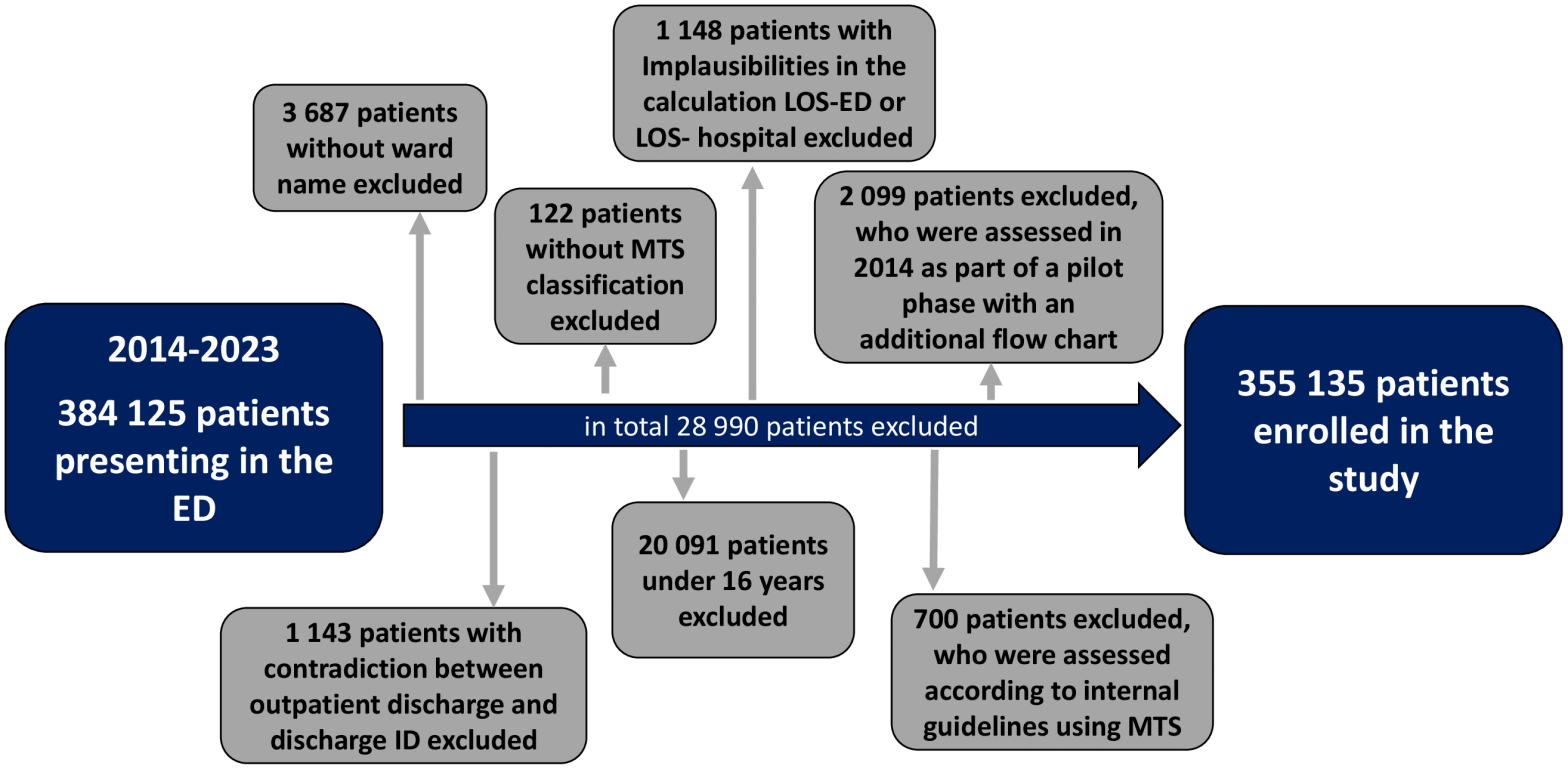
Exclusion criteria of the study. The chronological order of the exclusions is reflected in the sequence.

Already admitted inpatients without a recorded ward name were excluded, as they could not be classified into a care level. In addition, patients were excluded if there was a contradiction in the system between outpatient discharge and discharge ID. Patients for whom the initial assessment protocol was terminated prematurely without a triage level were also excluded. Patients younger 16 years of age were also excluded. Any implausibility in the length of stay in emergency department (LOS-ED) calculation or length of stay in hospital (LOS- hospital), e.g., duration ≤ 0 minutes, was not taken into account. Due to an internal clinical agreement with colleagues in the ENT department (foreign body in the nose), an artificial higher classification was made for a small collective. As this application was not MTS-compliant, this patient group was also excluded. In addition, patients who were assessed with an additional flowchart as part of a pilot phase in 2014 were excluded (Fig 1).

### Statistics and frequency definitions

Statistical analysis was carried out using R version 4.4.2 (R Core Team 2024: R: a language and environment for statistical computing, R Foundation for Statistical Computing, Vienna, Austria). Descriptive analysis included the calculation of mean values and standard deviations with ranges for continuous variables and frequencies (absolute and relative) for categorical variables. The following variables were analyzed descriptively: sex, age, patient allocation (walking or by ambulance), urgency level, patient admission (outpatient, normal ward, ICU), presentational flow chart and discriminator question. Additionally, the distributions of variables are presented graphically by boxplots and bar plots, as appropriate.

Short-term mortality and 30-day mortality based on the length of stay in hospital of admitted patients across the five MTS urgency levels were analyzed using Kaplan-Meier curves (deriving estimates and 95% confidence intervals of 12-hour and 30-day survival probabilities).

The accuracy of the MTS, i.e., the ability to predict admission and in-house mortality was assessed via the area under the curve (AUC) of the receiver operating characteristic (ROC) curves and their 95% confidence intervals (CI). For this, level of care was treated as an ordinal variable.

In particular, the following surrogate parameters were used to determine the ROC:

- inpatient admission
- admission to the intensive care unit (ICU)
- death within 12 hours/ within 30 days of initial assessment [6,10]

### Ethics statement

The study received approval (No. 2024–203-BO) from the chairman of the local ethics committee (K. Racké, MD, PhD, professor, University Bonn). The Ethics Committee for Clinical Trials on Humans and Epidemiological Research with Personal Data of the Medical Faculty of the Rheinische Friedrich-Wilhelms-Universität Bonn confirms that, in accordance with §15 of the professional code of conduct for physicians, there are no professional or ethical concerns to be raised [11]. Furthermore, following consultation with the responsible data protection officer, as stipulated by German data protection regulations, the physician may use existing patient data for analyses without explicitly asking for the consent of patient. All clinical data collected and evaluated in the current study were pseudonymized prior to analysis. The authors had no access to information during and after data collection that could identify individual participants. The study design is consistent with the Declaration of Helsinki [12].

## Results

### Baseline

During the observation period of ten years (2014–2023), a total of 355,135 emergency patients were included in the big data analysis. Viewed longitudinally, there was a continuous increase in the number of emergency patients requiring care in the emergency department. In 2023, 9022 more emergency patients (29,9%) were treated than in 2014 (Table 1). The sex distribution in the observation period does not show statistically relevant fluctuations. In contrast to the national average (♀ > ♂), males slightly outnumbered females among emergency patients in all observation years (Table 1). In terms of age, a continuous, clear increase in the mean value can be seen. The average age was 48.1 years in 2014 and 52.1 years in 2022 (Table 1).

**Table 1:** Baseline characteristics.

	2014	2015	2016	2017	2018	2019	2020	2021	2022	2023
	(N = 30156)	(N = 32853)	(N = 33695)	(N = 33988)	(N = 34168)	(N = 37302)	(N = 35458)	(N = 38269)	(N = 40070)	(N = 39176)
Sex										
Male	15779 (52.3%)	17020 (51.8%)	17491 (51.9%)	17604 (51.8%)	17658 (51.7%)	19174 (51.4%)	18431 (52.0%)	19717 (51.5%)	20895 (52.1%)	20454 (52.2%)
Female	14368 (47.6%)	15826 (48.2%)	16197 (48.1%)	16381 (48.2%)	16504 (48.3%)	18116 (48.6%)	17024 (48.0%)	18549 (48.5%)	19168 (47.8%)	18709 (47.8%)
Missing	9 (0.0%)	7 (0.0%)	7 (0.0%)	3 (0.0%)	6 (0.0%)	12 (0.0%)	3 (0.0%)	3 (0.0%)	7 (0.0%)	13 (0.0%)
Age										
Mean (SD)	48.1 (20.6)	49.4 (21.0)	49.4 (21.0)	50.1 (21.2)	50.9 (21.2)	50.8 (21.2)	51.4 (21.3)	52.1 (21.5)	52.1 (21.7)	51.7 (21.4)
[Min, Max]	[16.0, 103]	[16.0, 105]	[16.0, 104]	[16.0, 102]	[16.0, 104]	[16.0, 103]	[16.0, 106]	[16.0, 109]	[16.0, 104]	[16.0, 103]
Missing	2 (0.0%)	1 (0.0%)	4 (0.0%)	4 (0.0%)	2 (0.0%)	2 (0.0%)	5 (0.0%)	3 (0.0%)	6 (0.0%)	8 (0.0%)
Allocation										
Walking	19209 (63.7%)	19816 (60.3%)	21775 (64.6%)	21471 (63.2%)	20809 (60.9%)	23509 (63.0%)	21642 (61.0%)	22072 (57.7%)	24406 (60.9%)	25460 (65.0%)
Emergencies/ Ambulance	6338 (21.0%)	8037 (24.5%)	7086 (21.0%)	7821 (23.0%)	8461 (24.8%)	8921 (23.9%)	9251 (26.1%)	11410 (29.8%)	10825 (27.0%)	8917 (22.8%)
Others	4609 (15.3%)	5000 (15.2%)	4834 (14.3%)	4696 (13.8%)	4898 (14.3%)	4872 (13.1%)	4565 (12.9%)	4787 (12.5%)	4839 (12.1%)	4799 (12.2%)
Urgency level										
Blue	1788 (5.9%)	1964 (6.0%)	2327 (6.9%)	2179 (6.4%)	2122 (6.2%)	2590 (6.9%)	2626 (7.4%)	2214 (5.8%)	2461 (6.1%)	2594 (6.6%)
Green	14762 (49.0%)	16402 (49.9%)	17248 (51.2%)	16992 (50.0%)	17050 (49.9%)	18618 (49.9%)	17471 (49.3%)	17179 (44.9%)	18397 (45.9%)	18416 (47.0%)
Yellow	9357 (31.0%)	9259 (28.2%)	9146 (27.1%)	9669 (28.4%)	9559 (28.0%)	9541 (25.6%)	8957 (25.3%)	11108 (29.0%)	11930 (29.8%)	11533 (29.4%)
Orange	3860 (12.8%)	4839 (14.7%)	4584 (13.6%)	4788 (14.1%)	5041 (14.8%)	6097 (16.3%)	5941 (16.8%)	7241 (18.9%)	6796 (17.0%)	6182 (15.8%)
Red	389 (1.3%)	389 (1.2%)	390 (1.2%)	360 (1.1%)	396 (1.2%)	456 (1.2%)	463 (1.3%)	527 (1.4%)	486 (1.2%)	451 (1.2%)
Patient admission										
Outpatient	21135 (70.1%)	22082 (67.2%)	23007 (68.3%)	22812 (67.1%)	22524 (65.9%)	24501 (65.7%)	22606 (63.8%)	23771 (62.1%)	26158 (65.3%)	26140 (66.7%)
Normal ward	7548 (25.0%)	8472 (25.8%)	8550 (25.4%)	8834 (26.0%)	9072 (26.6%)	9560 (25.6%)	9354 (26.4%)	10557 (27.6%)	10210 (25.5%)	9658 (24.7%)
Intensive care unit	1473 (4.9%)	2299 (7.0%)	2138 (6.3%)	2342 (6.9%)	2572 (7.5%)	3241 (8.7%)	3498 (9.9%)	3941 (10.3%)	3702 (9.2%)	3378 (8.6%)

### Patient allocation

The group of walking emergency patients accounted for the largest proportion in all observed years, followed by patients arriving by ambulance. In 2021, the year impacted by the coronavirus pandemic, patient allocation by paramedics and emergency physicians accounted the highest percentage of emergency cases in the observed decade, whereas walking-in patients constituted the lowest percentage (Table 1).

### MTS urgency level

**Fig 2:**
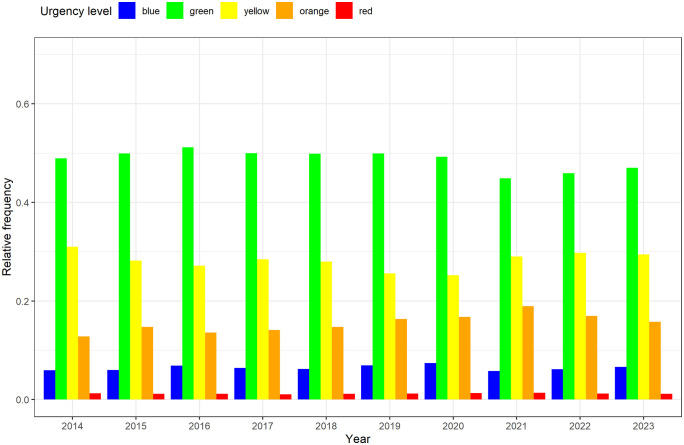
Frequency distribution of MTS urgency levels in the five MTS levels.

The proportion of “green” patients is highest in all years, followed by the “yellow”, “orange”, “blue” and “red” urgency levels. There is a noticeable change in the ratio in the coronavirus years 2020/2021, where the percentage of “green” emergency patients falls to a minimum, accompanied by an increase in the relative frequency of “orange” and “yellow” emergency patients (Table 1 and Fig 2).

**Fig 3:**
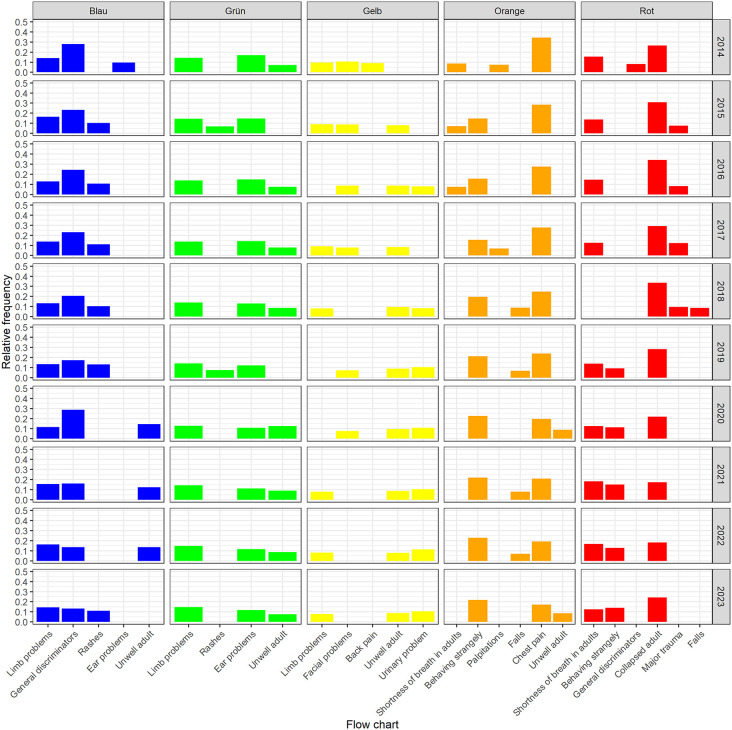
Top three presentational flow charts within the five different urgency levels.

The top three flow charts in the longitudinal progression over the ten years are shown in Fig 3. Overall, the presentational flowcharts appear to be stable over a 10-year period. For example, the presentational flow chart “limb problems” was almost always among the top three in the lower urgency classifications (“yellow”, “green”, “blue”), as was the presentational flow chart “unwell adult”. In the higher urgency levels (“orange” and “red”), it was the presentational flow charts “chest pain”, “shortness of breath in adults” and “collapsed adult”.

**Fig 4:**
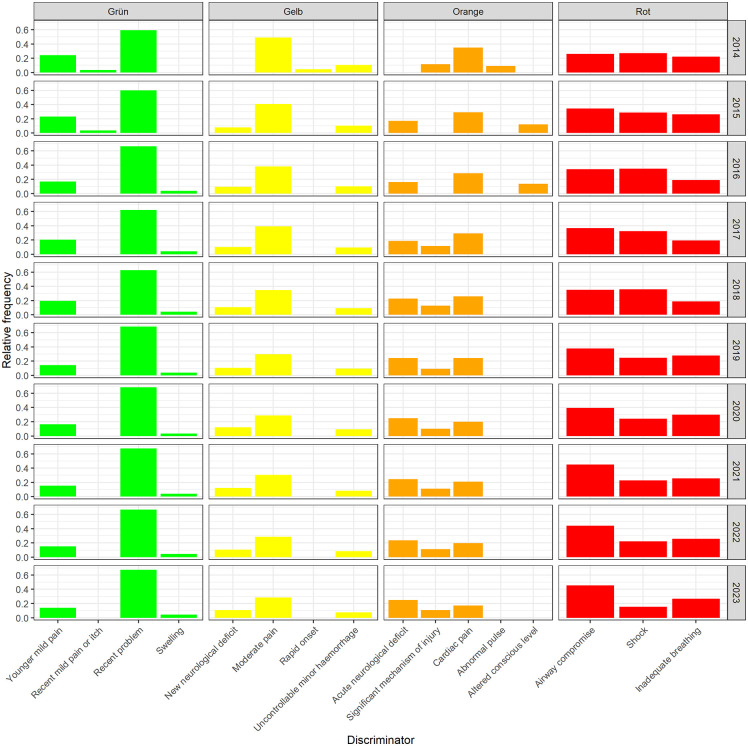
Top three discriminator questions within the five different urgency levels.

At the discriminator level, stability over the observation period was also observed. In the green triage category, “recent problem” was the most frequently used discriminator over the decade, while “moderate pain” predominated in the yellow triage category. Notably, the same discriminators were consistently applied in the red triage category throughout the 10-year period. The top three discriminator questions in the longitudinal progression are shown in Fig 4.

### Patient admission

**Fig 5:**
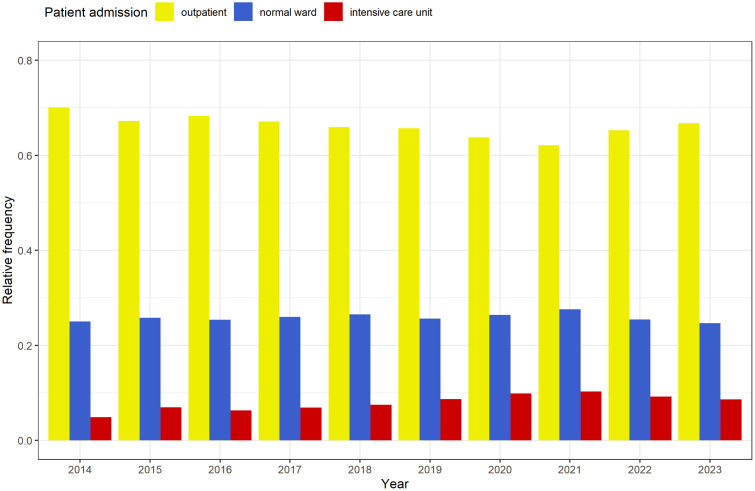
Frequency distribution of the types of patient admission (outpatient, normal ward, intensive care unit).

Overall, the distribution of the different care levels (outpatient, normal ward, intensive care unit) of emergency patients remained stable over the observed ten years. Once again, the influence of the pandemic in 2021 is striking, with fewer outpatients and a higher rate of inpatient admissions and ICU admissions (Table 1 and Fig 5).

**Fig 6:**
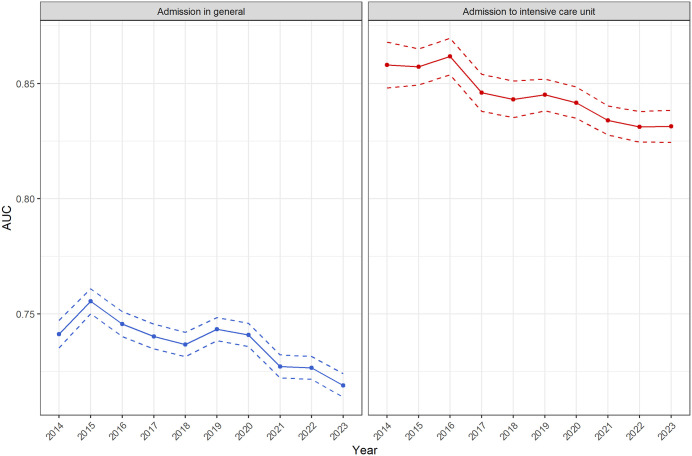
AUC values from ROC analysis for the decade 2014–2023 in terms of “admission in general” and “admission to intensive care unit”.

Over the entire period, the AUC of hospital admission in general was about 0.1 above the AUC admission to ICU. In both cases, the prediction of admission in terms of sensitivity and specificity across the five MTS levels showed no relevant differences. For the AUC of hospital admission in general, the value ranged from 0.756 (95% CI 0.750–0.761) to 0.719 (95% CI 0.714–0.724), and for the AUC admission to ICU, it ranged from 0.862 (95% CI 0.854–0.870) to 0.831 (95% CI 0.824–0.838) (Table 2 and Fig 6).

**Table 2:** AUC values from ROC analysis for the decade 2014–2023 in terms of “admission in general” and “admission to intensive care unit”.

	Admission in general		Admission to ICU	
year	AUC	95%-CI	AUC	95%-CI
2014	0.741	0.735-0.747	0.858	0.848-0.868
2015	0.756	0.750-0.761	0.857	0.849-0.865
2016	0.746	0.740-0.751	0.862	0.854-0.870
2017	0.740	0.735-0.746	0.846	0.838-0.854
2018	0.737	0.731-0.742	0.843	0.835-0.851
2019	0.743	0.738-0.748	0.845	0.838-0.852
2020	0.741	0.736-0.746	0.842	0.835-0.848
2021	0.727	0.722-0.732	0.834	0.828-0.840
2022	0.727	0.722-0.732	0.831	0.825-0.838
2023	0.719	0.714-0.724	0.831	0.824-0.838

AUC= Area under the curve, 95%-CI= 95% confidence interval

### Mortality

**Fig 7:**
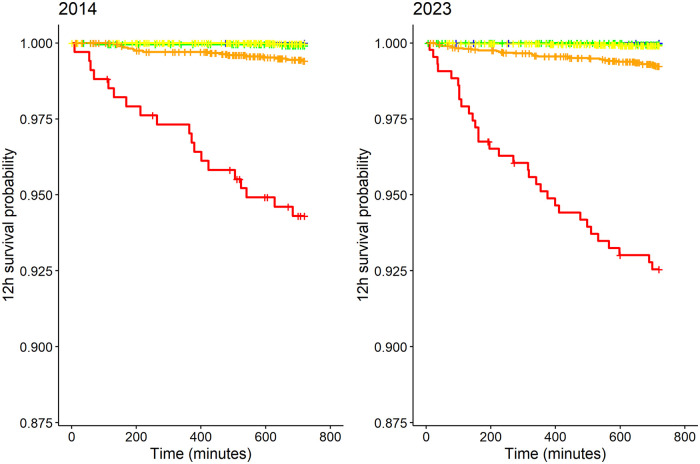
Kaplan-Meier curve in terms of “12-hour survival probability” comparing 2014 and 2023.

**Fig 8:**
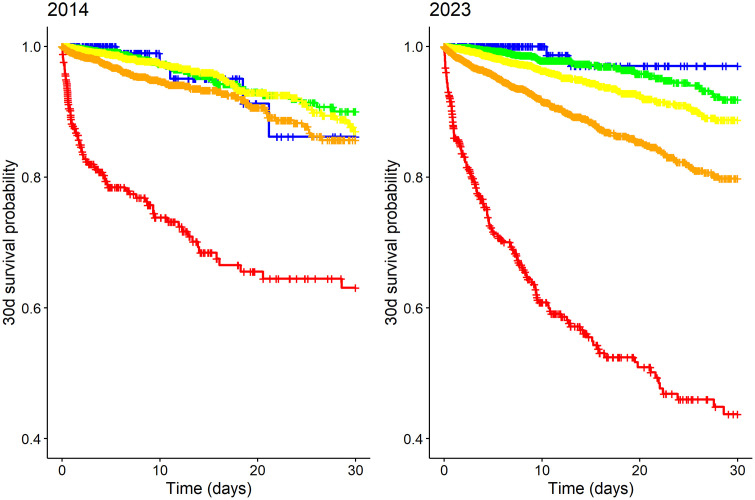
Kaplan-Meier curve in terms of “30-day survival probability” comparing 2014 and 2023.

**Fig 9:**
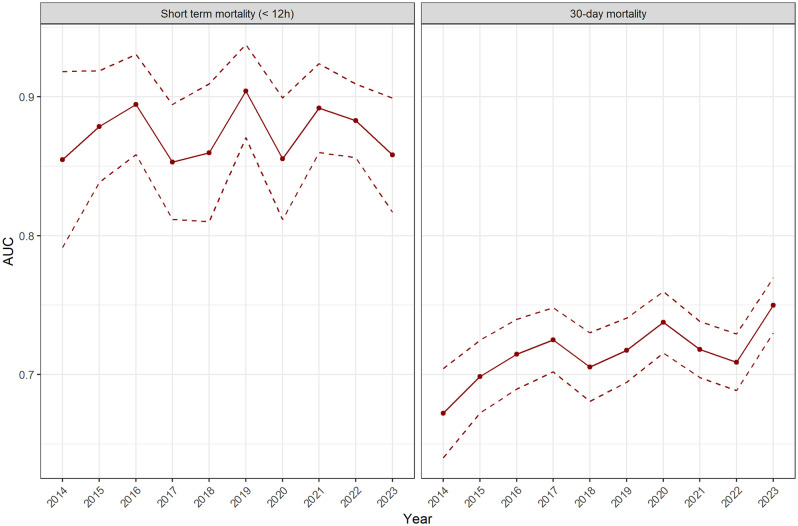
AUC values from ROC analysis in the decade 2014–2023 in terms of “short-term mortality (< 12 hours)” and “30-day mortality”.

The Kaplan-Meier analyses showed that the 12-hour survival probabilities in the “blue”, “green”, “yellow” and “orange” stages were each above 0.99 (95% CI 0.989–1.0) and in the red group, above 0.89 (95% CI 0.869–0.968) (Fig 7). The prediction of short-term mortality (AUC) ranged from 0.845 (95% CI 0.838–0.852) in 2019 to 0.894 (95% CI 0.858–0.931) in 2016 (Table 3). The pattern was different regarding 30-day mortality. While the results for the “blue” to “orange” stages show no abnormalities (estimated 30-day survival probabilities consistently > 0.8), the 30-day survival probability in the “red” stage decreased from 0.63 (95% CI 0.560–0.710) (2014) to 0.44 (95% CI 0.372–0.541) (2023) (Table 4 and Fig 8). The prediction of 30-day mortality (AUC) increased from 0.672 (95% CI 0.640–0.704) (2014) to 0.750 (95% CI 0.730–0.770) (2023) (Table 3 and Fig 9).

**Table 3:** AUC values from ROC analysis in the decade 2014–2023 in terms of “short-term mortality (< 12 hours)” and “30-day mortality”.

	12-hour mortality		30-day mortality	
**year**	**AUC**	**95%-CI**	**AUC**	**95%-CI**
**2014**	0.855	0.791-0.918	0.672	0.640-0.704
**2015**	0.879	0.838-0.919	0.699	0.672-0.725
**2016**	0.894	0.858-0.931	0.715	0.690-0.740
**2017**	0.853	0.812-0.894	0.725	0.702-0.748
**2018**	0.860	0.810-0.909	0.705	0.681-0.730
**2019**	0.845	0.838-0.852	0.743	0.738-0.748
**2020**	0.855	0.811-0.899	0.738	0.716-0.760
**2021**	0.892	0.860-0.924	0.718	0.698-0.738
**2022**	0.883	0.856-0.909	0.709	0.688-0.729
**2023**	0.858	0.817-0.899	0.750	0.730-0.770

AUC= area under the curve, 95%-CI= 95% confidence interval

**Table 4:** Death in the decade 2014–2023 in terms of “short-term mortality (< 12 hours)” and “30-day mortality” in the urgency levels “red” and “orange”.

year	MTS level	12-hour survival	95%-CI	30-day survival	95%-CI
**2014**	orange	0.994	0.991-0.997	0.857	0.822-0.893
	red	0.943	0.918-0.968	0.631	0.560-0.710
**2015**	orange	0.994	0.991-0.996	0.821	0.789-0.855
	red	0.903	0.873-0.934	0.611	0.549-0.679
**2016**	orange	0.992	0.989-0.995	0.788	0.752-0.827
	red	0.904	0.875-0.935	0.524	0.455-0.604
**2017**	orange	0.993	0.990-0.996	0.820	0.787-0.854
	red	0.914	0.885-0.944	0.544	0.478-0.618
**2018**	orange	0.994	0.992-0.997	0.847	0.820-0.875
	red	0.924	0.898-0.951	0.527	0.468-0.594
**2019**	orange	0.995	0.993-0.997	0.829	0.802-0.858
	red	0.910	0.883-0.937	0.560	0.500-0.627
**2020**	orange	0.995	0.993-0.997	0.821	0.792-0.851
	red	0.922	0.898-0.947	0.530	0.469-0.599
**2021**	orange	0.994	0.992-0.996	0.805	0.777-0.834
	red	0.896	0.869-0.923	0.517	0.463-0.577
**2022**	orange	0.991	0.989-0.994	0.789	0.760-0.820
	red	0.902	0.875-0.930	0.448	0.382-0.525
**2023**	orange	0.992	0.990-0.995	0.798	0.769-0.828
	red	0.925	0.901-0.951	0.438	0.372-0.514

## Discussion

This is one of the first longitudinal studies to evaluate the Manchester Triage System in an emergency department over a ten-year period using a large dataset. However, direct comparison with other large-scale studies including several hundred thousand patients is limited due to differences in the observation period and the specific study focus [13].

The current study focuses on the absolute accuracy of the MTS and on its performance over a period of 10 years, taking exogenous influences into account.

Surprisingly, the AUC values of the first validation work on the German MTS (observation period 2010/2011) could be confirmed with the present work. The AUC determined at the time for the “hospital admission in general” group was 0.749 and for the “admission to the intensive care unit” group 0.871 [5].

When considering recent publications from 2025, it becomes apparent that the AUC values they reported are almost identical to those we measured over the ten-year period. In their single-centre study from Merano, the AUCs were 0.733 for hospitalisation and 0.862 for ICU admission [14]. In contrast, another study determined an AUC of 0.719 for the MTS in predicting for “admission to the intensive care unit” in a large, unselected population of medical ED patients [15].

As described, the parameter ‘ICU admission’ is used as a surrogate for evaluating the MTS, and potential bias due to contextual factors, such as limited ICU capacity, cannot be entirely excluded. In the current study, however, this is negligible, as hospital policy mandates that all patients requiring intensive care be admitted to the ICU within one hour—a procedure that is followed in nearly all cases.

Despite the good AUC values, the scientific goal must be to further improve the MTS. Initial data from our own studies (publication forthcoming) show, based on univariate and multivariate regression analyses, that there are certain risk constellations that are associated with significantly increased mortality. One factor, for example, is the age of the emergency patients. Since age is not explicitly considered in the individual MTS flow charts, future triage may benefit from decision-support systems based on machine learning algorithms to incorporate this factor and others, such as comorbidities, into risk assessment. The aim is not to replace medical professionals as decision-makers, but to provide them with relevant information and support them in their decision-making – without fatigue, even when they have a high workload [16].

Another important aspect is the prediction of inpatient admission, particularly with regard to intensive care capacity planning. With an average AUC of 0.84, the ICU could be notified during triage of the potential need for an intensive care bed in patients assigned to the highest triage categories. This may substantially improve patient flow and resource management in the emergency department. In addition to the absolute AUC values, the values in the longitudinal course are impressive, showing remarkable stability over a decade. In our opinion, this result can be seen in connection with the user’s adherence to the triage system. Bürger et al. [1] were able to show in a recent study that inaccurate results in urgency classification were largely due to user error.

Another publication from 2025 investigated the accuracy of nonspecific presentational flow charts in a paediatric emergency department. The authors also concluded that poorer triage quality can be explained by a lack of compliance with the triage system on the part of nursing staff [17]. Since the introduction of the MTS, the ED at Bonn University Hospital has placed great importance on triage auditing. The authors are convinced that the audits carried out two to three times a year have led to consistent figures over a decade.

Also of interest are findings on factors influencing the application and accuracy of the MTS [13]. By comparing three different hospitals, these authors identified varying levels of accuracy in the application of the MTS. One factor shown to affect performance was the characteristics of the patient population.

It is known that population characteristics, including demographics and disease severity, influence the performance of diagnostic tests [18]. In the case of triage, higher patient complexity is expected to contribute to lower performance of a triage system, as patients with rare diseases or multiple comorbidities are more difficult to triage [19]. The prevalence of diseases may also play a role, so that the triage course tends to have lower urgency levels at higher prevalence (habit effect) [18]. Two exogenous factors with a potential influence on the triage system can be derived directly from the results of the current study. The first is the demographic trend from 2014 to 2023 with the average age increasing from 48.1 years to 51.7 years over the ten-year period. This increase in the average age is even greater than the nationwide average age increase in the same period. Consequently, the disease severity also increased during the observation period. Most recently, the Global Burden of Disease Study collected information on 306 diseases in 188 countries, concluding that living longer often means suffering longer. The WHO comes to the same conclusion, that often a long life is also accompanied by considerable impairment due to several incurable diseases [20–22]. On the other hand, the effects of Covid-19 pandemic in 2020/2021 are reflected in reduced patient presentations and proportionately higher admissions via emergency services, e.g., ambulances. At the same time, there was a peak in admissions to the ICU during the pandemic phase. Our figures are comparable to the data from a multicenter, Germany-wide data collection in emergency departments [23].

In our opinion, two other exogenous factors can be seen as influencing the MTS. One is the employee turnover recorded over the observation period. A reliable, albeit unfortunate, marker for the high staff turnover is the fact that today only a few employees from 2014 are working in the UKB ED.

The second exogenous factor relates to the changes in healthcare policy in emergency care over the observation period. The steady increase in the number of patient contacts, except for the corona pandemic, is due to hospital closures during the last ten years and high demand in general practitioner (GP) surgeries [24,25].

In summary, it is evident that the MTS was subject to significant exogenous influences during the ten-year study period. As a result, it is remarkable that the data show exceptional stability over the entire period and do not contain a single outlier. In this respect, the results of Zachariasse et al. [3] cannot be reconciled with our data. Even the pandemic influences, with the highest patient allocation by paramedics and emergency physicians, an increase in “orange” and “yellow” emergency patients, and the highest rate of ICU admissions, do not result in any significant change in accuracy. In our opinion, this once again underlines the need to constantly train new employees, i.e., system users, in MTS.

Nevertheless further prospective multicenter studies are needed. Only then will it be possible to include different clinical settings, patient populations, and validation methods. Emergency department sizes, populations, and practices vary worldwide, yet triage must be performed in all settings. Future research should therefore focus on large multicenter studies across diverse hospitals.

Evaluation of the presentational flow charts used over the longitudinal course is also interesting. Even if the selection by the nurse is directly related to the leading symptom presented by the patient, i.e., the level of care provided by the hospital, the frequency of use of individual presentational flow charts or discriminators is remarkable. Our results are in line with the observations made in three different hospitals with a different case mix [13]. The most frequently used presentational flow charts were “limb problems”, “unwell adult”, “abdominal pain in adults”, “chest pain”, “shortness of breath in adults” and “headache”, which together accounted for 39% of all presentational flow charts used in adult patients.

The use of mortality to assess the accuracy of a triage system is certainly the “hardest” outcome parameter and, in the opinion of the authors, must be considered in a highly differentiated manner. The use of 30-day mortality has the disadvantage that confounders (e.g., nosocomial pneumonia) can have a relevant influence on survival during hospitalization. In our opinion, this is also reflected in the data from a systematic review. The authors showed in their review that most studies had a high sensitivity (> 90%) of the triage systems for identifying patients with a high mortality risk (death in the ED). However, the sensitivity was low (< 80%) for the identification of patients with critical illness and patients who died within a few days of the ED visit or during hospitalization [26]. Our data confirm the key findings of the aforementioned study: among patients assigned to low-urgency triage levels (“yellow,” “green,” and “blue”), no deaths occurred within 12 hours. The individual analysis of deceased patients in the “green” and “blue” triage levels at that time showed that these were exclusively emergency patients who were in an “end of life” situation (e.g., terminal HIV) and whose low urgency classification was completely correct. The prediction of short-term mortality (< 12 hours) in our current study is at a very high level with AUC values between 0.845 and 0.894. The prediction of 30-day mortality (AUC), on the other hand, is between 0.672 and 0.750. If we apply this fact, i.e., the correct admission of “green/blue” emergency patients, to the prediction of inpatient admission and thus to the accuracy of the MTS, we can actually assume a slightly better accuracy. A closer analysis shows that mortality has increased over the ten-year observation period, particularly within the “red” and “orange” triage categories, while the 30-day survival probability has progressively declined. As already discussed above, demographic developments likely contribute to rising disease severity and comorbidity. In principle, two additional explanations are conceivable. First, alongside the supraregional trauma centre, two further specialised centres were established at the UKB over the course of the decade: the Cardiac Arrest Center and the neurovascular network. Consequently, more patients are now admitted who, by definition, present with high initial mortality risk – such as resuscitated patients and stroke patients – leading to increased 30-day mortality and, correspondingly, reduced 30-day survival. This interpretation is supported by the steadily rising number of “orange” patients and the increasing number of ICU admissions. As illustrated in the Kaplan–Meier curves, differences in survival probability only become evident after two to three days. Second, a deterioration in the intensive care provided to these emergency patients cannot be ruled out. Without further detailed analysis of specific ICU data, this consideration must remain hypothetical and requires investigation in future studies.

When focusing specifically on patients triaged as “red”, a shift in case types can be observed over the decade, with a decrease in polytrauma patients and an increase in cardiac arrest patients. It should be noted that patients with cardiac arrest inherently have a higher mortality risk than trauma patients. Fig 3 illustrates this trend, which may account for the observed decline in 30-day survival despite the stable overall number of “red” patients.

### Strength and limitations

The strength of the current study lies in the large cohort of almost 360,000 emergency patients.

One limitation of our study is that it is a monocentric study with retrospective data analysis. However, since the study was conducted on a cohort from a tertiary care provider a comprehensive spectrum of diseases and injury patterns was included in the evaluation. Also, there were several version changes of the MTS during the observation period. However, we consider this influence negligible with regard to the validity of our work, particularly since the changes mainly involved the introduction of new paediatric presentational flow charts. Previous studies have shown that modifications between MTS versions only minimally affect the results [13].

## Conclusion

This longitudinal large-scale study demonstrates that the Manchester Triage System (MTS) provides a consistently reliable initial assessment of emergency department patients over a ten-year period, even under substantial exogenous influences such as demographic change, health policy changes, staff turnover, and the COVID-19 pandemic.

Despite its overall good discriminatory performance, the results also indicate that further refinement of the MTS is warranted. Increasing patient complexity, ageing populations, and changing patterns of comorbidity may limit the predictive capacity of symptom-based flow charts alone. Future developments should therefore focus on integrating additional risk factors, such as age and comorbidities, potentially supported by artificial intelligence, to enhance risk stratification and resource prediction without replacing clinical decision-making.

Overall, the findings underline that the MTS is a robust and clinically useful triage system, but that continuous staff training, regular auditing, and further system development are essential to maintain and improve triage quality and to optimize emergency department workflow and patient safety.
